# CD73 Is a Major Regulator of Adenosinergic Signalling in Mouse Brain

**DOI:** 10.1371/journal.pone.0066896

**Published:** 2013-06-12

**Authors:** Natalia Kulesskaya, Vootele Võikar, Marjaana Peltola, Gennady G. Yegutkin, Marko Salmi, Sirpa Jalkanen, Heikki Rauvala

**Affiliations:** 1 Neuroscience Center, University of Helsinki, Helsinki, Finland; 2 Department of Biosciences, University of Helsinki, Helsinki, Finland; 3 MediCity and Department of Medical Microbiology and Immunology, University of Turku and National Institute of Health and Welfare, Turku, Finland; 4 Department of Medical Biochemistry and Genetics, University of Turku, Turku, Finland; University of Colorado Denver, United States of America

## Abstract

CD73 (ecto-5’-nucleotidase) is a cell surface enzyme that regulates purinergic signalling by desphosphorylating extracellular AMP to adenosine. 5′-nucleotidases are known to be expressed in brain, but the expression of CD73 and its putative physiological functions at this location remain elusive. Here we found, using immunohistochemistry of wild-type and CD73 deficient mice, that CD73 is prominently expressed in the basal ganglia core comprised of striatum (caudate nucleus and putamen) and globus pallidus. Furthermore, meninges and the olfactory tubercle were found to specifically express CD73. Analysis of wild type (wt) and CD73 deficient mice revealed that CD73 confers the majority of 5’-nucleotidase activity in several areas of the brain. In a battery of behavioural tests and in IntelliCage studies, the CD73 deficient mice demonstrated significantly enhanced exploratory locomotor activity, which probably reflects the prominent expression of CD73 in striatum and globus pallidus that are known to control locomotion. Furthermore, the CD73 deficient mice displayed altered social behaviour. Overall, our data provide a novel mechanistic insight into adenosinergic signalling in brain, which is implicated in the regulation of normal and pathological behaviour.

## Introduction

CD73 (also known as ecto-5’-nucleotidase) is a key regulator of the extracellular nucleotide breakdown. Extracellular AMP can be hydrolyzed to adenosine by CD73, or converted to ADP by adenylate kinase. Adenosine has multiple signalling functions in many different tissues, since it can bind to four different receptors, A1, A2A, A2B and A3 [1].

In the central nervous system (CNS), adenosine plays a critical role in controlling a multitude of neural functions [2]–[4]. Through the activation of its G-protein coupled receptors, adenosine is involved in diverse physiological and pathological processes such as in the regulation of sleep, general arousal state and activity, local neuronal excitability, and coupling of the cerebral blood flow to the energy demand. Moreover, manipulation of adenosine signalling may have therapeutic potential in neurodegenerative diseases such as Alzheimer’s disease, Parkinson’s disease and Huntington’s disease, and in psychiatric diseases such as schizophrenia and autism [5]. Therefore, alterations in the adenosine concentrations could have dramatic effects on functions and behavior of the whole organism [6], [7].

In contrast to the wealth of information on the role of adenosine in the central nervous system, almost nothing is known about the functions of CD73 at that location. Importantly, there are altogether seven different 5’-nucleotidases in man [8], and therefore the contribution of CD73 to the adenosine production can basically only be dissected using gene-deficient mice. CD73 has been shown to regulate the vascular permeability and inflammatory balance in multiple organs, including inflamed brain [9]. Adenosine, in contrast to ADP (and ATP), is an anti-inflammatory and a leakiness inhibiting molecule, and CD73 has been shown to mediate its cell migratory and vascular barrier functions mainly via its enzymatic activity, although other mechanisms may be involved as well [10]–[12].

The role of CD73 in controlling behavioural aspects has remained unknown. Here we used CD73 deficient mice to dissect the contribution of CD73 to enzymatic activity of the purinergic signalling cascades in brain. Moreover, we performed comprehensive behavioural phenotyping of these mice using both traditional tests and automated monitoring in natural home-cage environment. The results reveal new functions for CD73 in controlling exploratory activities and social interactions.

## Materials and Methods

All experiments have been carried out in accordance with the Guidelines laid down with the European Communities Council Directive of 24 November 1986 (86/609/EEC) and were approved by the County Administrative Board of Southern Finland (license number ESLH-2007-09104/Ym-23).

### Animals

CD73 deficient mice were generated and back-crossed for 9 generations to C57/B16/J strain as described [12]. The CD73 deficient and the wild type B6 background strain (wt) mice were identified using one PCR reaction for the wt allele, and one for the recombined allele, as described.

Twenty-nine CD73 deficient mice (16 females, 13 males) and 38 wt (23 females, 15 males) were used for the primary behavioural phenotyping essentially as described [13], [14]. Additional 17 mice (9 CD73 deficient and 8 wt; all females) were used for the IntelliCage assays. Animals were kept under standard conditions (group-housed 2–5 mice per cage in a mixed sex colony) with a 12 h/12 h lights on-off time (lights on at 6 p.m.), relative humidity 50–60%, and room temperature 21+/− 1°C. Food and water were available *ad libitum*. The mice were group-housed (2–5 animals per cage) Behavioural testing began at the age of 8 weeks. All experiments (with exception of IntelliCage experiments) were carried out between 9 a.m and 2 p.m.

### Immunohistochemistry

Mice were anesthetized with pentobarbital and perfusion fixed with 4% paraformaldehyde (PFA). Brains were immersion fixed overnight, cryoprotected in 30% sucrose and freezed on dry ice. Floating sections (40 µm) were blocked with 2% bovine serum albumin (BSA) and 0.5% Triton X-100 in PBS, and incubated with rat antibody to CD73 (clone TY/23, BD Biosciences) for 48 h (5 µg/ml), followed by Alexa Fluor 488 conjugated goat anti-rat antibody (2 µg/ml, Invitrogen) and Alexa Fluor 488 conjugated donkey anti-goat antibody (2 µg/ml, Invitrogen). Antibody dilutions were made in 2% bovine serum albumin (BSA) and 0.1% Triton X-100 in PBS. Thorough washings with 0.1% Triton X-100 in PBS were performed after each antibody incubation.

### Enzymatic assays

Brains were rapidly dissected from the wt and CD73 deficient mice, divided to forebrain, middle brain and cerebellum fractions, excised and incubated with a lysis buffer (0.2% Triton X-100 in PBS). After a 2-hour incubation at 4°C, the lysates were centrifuged for 10 min at 15000 g and the supernatants stored at –70°C. Total protein concentrations in the lysates were determined by Protein Assay Kit (Pierce, Rockford, IL) according to manufacturer’s instructions.

The standard enzyme assay was performed at 37°C in a final volume of 80 µl RPMI-1640 medium containing 4–6 µg brain lysate, 4 mmol/L β-glycerophosphate, various unlabelled nucleotides and tracer [2,8-^3^H]ATP (Perkin Elmer), [2,8-^3^H]ADP (Perkin Elmer) or [2-^3^H]AMP (Amersham Biosciences) as appropriate substrates. Purinergic activities were determined in the following ways: (i) for ATPase, ADPase and 5′-nucleotidase (AMPase) assays, brain lysates were incubated for 60 min with 400 µmol/L [^3^H]ATP, [^3^H]ADP or 300 µmol/L [^3^H]AMP, respectively; (ii) for detecting adenylate kinase, samples were incubated for 45 min with 400 µmol/L [^3^H]AMP in the presence of 750 µmol/L γ-phosphate-donating ATP. Catalytic reactions were terminated by applying aliquots of the mixture onto Alugram SIL G/UV_254_ sheets (Macherey-Nagel, Duren, Germany). Radiolabelled nucleotides and nucleosides were separated by TLC and quantified by scintillation β-counting, as described [15].

### Individual behavioural tests

The tests in elevated plus maze, open field, light-dark exploration, Y-maze, hot plate, rota-rod, pre-pulse inhibition, fear conditioning, water maze and forced swim test [13], [14] and in olfaction [16] were carried out essentially according to the previously described procedures. The previously described order of individual tests was followed in the behavioural screen [14].

#### Video tracking

During the elevated plus-maze, Y-maze, water maze, forced swim test and sociability test the paths of the mice were video-tracked by using a Noldus EthoVision 3.0 system (Noldus Information Technology, Wageningen, The Netherlands). The system recorded the distance travelled by the subjects, the time spent in pre-defined zones and the status of specified event recorder keys on the keyboard. The raw data were analysed by the same software.

#### Elevated plus maze

This test was used for assessment of unconditioned anxiety-like behaviour. The maze consisted of central platform (5×5 cm), two open (30×5 cm) and two closed arms (30×5×15 cm) with transparent side- and end-walls, and it was raised to 38.5 cm above the floor. The mouse was placed in the center of the maze facing one of the enclosed arms and observed for 5 min. The following parameters were measured: the time spent in the closed and open arms, number of entries to the open and closed arms, latency to the first entry to the open arm, the distance travelled and the number of rearings.

#### Open field activity

Activity was measured in the Activity Monitor system (MedAssiociates, St. Albans, VT). The mice were released in the corner of the open field arena (30×30 cm) facing the wall. Horizontal and vertical activities were recorded during 30 min. For the analyses, the arena was divided to a central (18×18 cm square) and a peripheral zone, and the following parameters were calculated: the distance travelled, the distance and the percent of the distance travelled in the zones, the time spent in both zones, the number of entries to the centre, latency to the fist entry to the centre, and the time spent in vertical activity.

#### Light-dark exploration

The experiments were performed in the Activity Monitor system modified with a dark box insert that is opaque to visible light and designed to cover half the area of the open field arena (an opening 5.5×7 cm allowed free movement between the compartments). Animals were placed to the open part of the arena and monitored for 10 min. The total distance travelled, the distance and percent of the distance travelled in light and dark zones, the time spent in zones, latency to entries to the dark zone and the time spent in vertical activity were calculated.

#### Y-maze

Spontaneous alternation performance was assessed in a symmetrical Y-maze under reduced light conditions (∼100 lx). Each arm was 30 cm long and 7 cm wide with transparent walls. Mice were allowed to explore the maze for 5 min, and the number and sequence of the arm entries were recorded. The percent of alternations was calculated as the number of alternations (entries into free different arms consecutively) divided by the total possible alternations (the number of total arm entries minus 2) and multiplied by 100 (e.g. visit sequence ABCCBAC contains 6 visits and 3 complete alternations, alternation 75%). In addition, the number of rearings, grooming behaviour and fecal boli were counted.

#### Hot plate

Standard hot plate (TSE Systems, Bad Homburg, Germany) was used for the assessment of nociceptive sensitivity. The plate was preheated to 52°C and the mouse was confined there by Plexiglas cylinder. Latency to show a hind paw response (licking or shaking) was taken as the pain threshold.

#### Rota-rod

The rota-rod test for motor coordination and motor learning was performed during 2 days and consisted of 3 subsequent trials per day with 1 h inter-trial intervals. Mice were placed on a slowly rotating drum (Ugo Basile, Comerio, Italy) that accelerated from 4 to 40 r.p.m. over a 5- min period. The latency to fall off was the measure of motor coordination, and improvement across the trials was the measure of motor learning.

#### Pre-pulse inhibition of acoustic startle reflex (PPI)

Experiments for loco-motor gaiting assessment were performed in Acoustic Startle Reflex System (Med Associates, St. Albans, VT). Animals were restrained in round acrylic holders and placed on a piezoelectric platform inside an isolated chamber. The background white noise was 65 dB. After a 5 min acclimatisation the test was performed in three blocks. The first block consisted of 5 white noise acoustic stimuli (SS, 105 dB, 40 ms) presented alone with an inter-trial interval of 8–15 s. The second block protocol included 50 trials of 5 different types. One of them was a startle stimulus (SS) alone as in block 1. In the four other trial types the startle stimulus was preceded by an acoustic pre-pulse (PPS, 20 ms) of 68, 72, 76 or 80 dB. The delay interval between PPS and SS was 10 ms. The third block was exactly the same as the first one. The startle response was averaged over 10 trials from block 2 for each trial type. The pre-pulse inhibition for each pre-pulse stimulus intensity was calculated using the formula: PPI = 100–[(PPS +SS startle response/SS alone startle response)×100].

#### Fear conditioning

A computer-controlled fear conditioning system (TSE Systems, Bad Homburg, Germany) was used for assessment of contextual and cue-dependent memory. The protocol consisted of 3 sessions: fear conditioning on the first day, the contextual memory test 24 h after the training followed by a cue-dependent memory test under new conditions after 2 h. The training was performed in a clear acrylic chamber with grid flow (4 mm diameter, 9 mm distance) within a fear conditioned box. Illumination was about 550 lx and loudspeakers provided a constant, white background noise (68dB). After a 2 min acclimatisation, a conditioned stimulus was applied for 30 s (CS, 10 kHz tone, pulsed 5 Hz, 75dB). The tone was terminated by a footstock (US, 0.7 mA, 2 s). The second CS-US pairing was applied after 30 s. After each test the animal chambers were cleaned by ethanol. Contextual memory was tested 24 h after the training in the same chamber and same conditions without any stimulation of tone or shock. The distance travelled and the total time of freezing were measured by infrared beams during 180 s. Cue-dependent memory was tested 2 h later in a novel context. The novel context was a similar size black Plexiglas chamber with a flat floor. Light was reduced to 50 Lx. Before each trial the chamber was cleaned with propanol. After 120 s of free exploration in the novel context, the CS was applied for another 120 s. The distance travelled and the freezing time was measured over the whole test period.

#### Water maze

The test was used for analyzing spatial learning and memory. Water maze consisted of a black circular swimming pool (120 cm diameter) and a black escape platform (10 cm diameter) submerged 0.5 cm under the water surface in the center of one of four imaginary quadrants. Mice were released to swim from random positions facing the wall, and the time to reach the escape platform was measured in each trial (maximum time allowed to swim was 60 s). Two training sessions consisting of three trials each were conducted daily. The interval between trials was 4–5 min and between the sessions about 3h. The platform was in the same place during the three training days (6 sessions), and was moved thereafter to an opposite quadrant for the next 2 days (4 sessions). The transfer tests were conducted on the next day after completing the training to the hidden platform and the moved hidden platform (after the 6th and 10th sessions). The animals were allowed to swim for 1 min in the maze without available platform. The spatial memory was estimated by the time spent in the training zone around the platform (30 cm diameter) and in the corresponding zones on three other quadrants, and by the time in the training quadrant and the number of entries to the training zones. Additionally, the swimming distance and the time spent within 10 cm from the wall (thigmotaxis) were measured. After the water maze with the hidden platform, one session was conducted with a visible platform. Time to reach the platform was measured.

#### Forced swim test

This method is used to estimate behavioural despair in a stressful and inescapable situation. The mice were placed for 6 min in the glass cylinder (18 cm diameter, 25 cm high) filled with 15-cm-deep water (23±1°C). The time of immobility (passive floating with only slight movements of the tail or one hind limb) was measured during the last 4 min of the test.

#### Olfaction

Olfaction was tested based on latency to sniffing and time spend in olfactory investigation of capsules with two different odors during 1 min trial. Cinnamon and cocoa were used as odors to be discerned in the test. Trials with cinnamon were repeated 4 times with 10 min interval. During the 5th dishabituation trial new odor (cocoa) was applied. The test was performed in a cage where the test mouse was acclimatizing for 20 min.

#### Assessment of barbering behaviour

Barbering behaviour was assessed by examining the hair and whisker loss in group-housed males. Barbering was scored as 0 ( =  intact hair), or 1 ( =  loss of hair).

#### Tube test

The tube test was chosen to measure social dominance in mice [17]. Two mice of the same sex and different genotype were placed in the opposite ends of a 30×3.5 cm transparent plastic tube and released simultaneously. The match ended, when one mouse completely retreated from the tube. The mouse remaining in the tube was the winner. Each animal was tested against all animals from the opposed group. The percent of lost matches as well as aggressive postures were scored for each animal. The matches, which lasted more than 2 min or in which the animals crossed over each other were not scored.

#### Resident-intruder test

The resident-intruder test was used to measure social activity and aggression in mice of both sexes. An intruder mouse (C57Bl/6J, same sex) was put in the cage, where the test mouse was acclimatizing for 30 min. The time spent in social activity (sniffing, hetero-grooming) and non-social activity (attack behaviour, digging, grooming and rearing) were recorded for 5 min.

#### Social novelty test

The social novelty test apparatus consisted of three rectangular compartments (18×35×18 cm) divided by Plexiglas walls with small openings allowing the animal to move between the compartments [18]. The test mouse was first allowed to habituate to the apparatus for 10 min. *Sociability test*: an unfamiliar mouse of the same sex (stranger 1) that had no prior contact with the test animal was placed in one of the side compartments. The location of the stranger 1 in either of the side compartments varied systematically between the trials. The stranger 1 mouse was enclosed in a small grid cage (7.5 cm diameter, 10 cm high) that allowed a snout contact between the bars but not biting or other fighting behaviour. The test mouse was then allowed to explore the whole apparatus for the next 10 min. The time spent in and entries into each compartment as well as the time spent sniffing near the unfamiliar mouse were recorded.

#### Social novelty preference

A second unfamiliar mouse (stranger 2) was placed in a cage in the chamber that was previously empty. The test mouse had a choice between an already-investigated mouse (stranger 1) and non-investigated unfamiliar mouse (stranger 2). Again, entries into the compartments as well as the time spent in each compartment and the time spent sniffing were recorded. The mice serving as unfamiliar ones had been earlier habituated to the test conditions. Strangers for the sociability and the social novelty tests were taken from separate cages.

#### Circadian activity

Using a Comprehensive Lab Animal Monitoring System (CLAMS; Columbus Instruments, USA), the animals were individually screened for their activity, food and water consumption for 72 h with the 12:12 light : dark cycle. Animals were placed to special individual cages with water and food available *ad libitum.* Data were automatically recorded every 30 min. Body weights were determined just before and after testing.

### Behavioural assessments in IntelliCage (IC)

IntelliCage (NewBehavior AG, Zurich, Switzerland) allows fully automated monitoring of spontaneous and learned behaviour of mice in the home cage environment without stress of social isolation and handling [19]. Female mice were placed into 2 cages (Tecniplast 2000, 37.5×55×20.5 cm) in a genotype-mixed order. Bedding material and plastic shelters were used as environmental enrichment. Usual food was available ad *libitum.* Each cage contained 4 operant corners with 2 openings allowing access to the nozzles of the drinking tubes. The openings could be blocked by motorized doors. Access into the corners was provided via a tubular antenna reading the transponder codes. Subcutaneously (in the dorso-cervical area) implanted RFID transponders (T-IS 8010 FDX-B, DATAMARS, Switzerland) were used for the subject identification in the operant corners of IntelliCage. The implantation of the transponders was done one week before the experiment started. The system measures the number and duration of the visits to every corner, nose-pokes to the door areas and licking of the drinking tubes. The following test schedule was applied: free adaptation (7 days), nosepoke adaptation (4 days), adaptation to drinking sessions (3 days), corner preference learning in sessions (4 days), reversal learning in sessions (3 days). All tests started between 11.00 –12.00 am.

#### Free adaptation

the mice were allowed to explore new environment with all gates open and free access to drinking tubes. Exploratory activity (corner visits for the first 1 h and before darkness), locomotor activity (corner visits) as well as circadian activity, anxiety parameters (latency to the first corner visit, nose-poke and licking), drinking behaviour (number of lickings, % of visits with licking) and spontaneous alternations were analysed.

#### Adaptation to nose-poke

All gates were closed at the beginning of module and mice were trained to poke into closed gates to reach drinking tubes. Only the first nosepoke of the visit opened the door for 7 s.

#### Adaptation to drinking session

gates were programmed to open after the first nose-poke only during two 1-hour periods, from 21:00 to 22:00 and from 02:00 to 03:00. Drinking sessions were applied for increasing the motivation to visit the corners and thereby providing defined time windows for testing learning.

#### Corner preference learning

during 4 days (8 sessions) the mice were trained to drink from only one corner. For each mouse a certain corner was programmed as the “correct” one where the first nosepoke of the visit was rewarded by opening the door and access to water during drinking sessions. One pair of mice (one wt and one CD73 deficient mouse) was assigned to each corner of the IntelliCage. Learning was measured as a percentage of visits to the “correct” corner.

#### Reversal learning

this test was used to assess the ability to relearn and extinct previously learned association. The mice had again access to water in one “correct” corner that was diagonally opposite to the previously used corner. Percentage of visits to the new “correct” corner and percentage of visits to the corner that was “correct” in the previous learning test were analysed.

#### Social behaviour

The last two nights of *Corner preference learning* and *Reversal learning* phases were chosen for assessment of social competition in the IntelliCage. The duration of visits in the correct corner during the first drinking session of the night was compared between wt and ko mice (8 pairs). In each mouse pair the animal that spent more time in the correct corner was designated as dominant.

### Real time qPCR

RNA was isolated from mouse brain using Trizol (Invitrogen) and cDNA was synthesized using a Reverse-iT kit (ABGene). Primers specific for A1R (forward) 5′-GTTTGGCTGGAACAACCTGA-3′, (reverse) 5′-ACACTTGATCACGGGCTCC-3 were used to determine gene expression levels and standardized to the GAPDH (forward) 5′-CCCCAATGTGTCCGTCGTG-3′, (reverse) 5′- GCCTGCTTCACCACCTTCT-3′ housekeeping gene using a SYBR-Green kit (ABGene) run on an ABI 7500 real time PCR system. To determine relative fold change (RFC), mRNA levels were normalized to the gene expression levels found in naïve wt mice, with 0.0 representing baseline values. Melt curve analyses were performed to measure the specificity for each qPCR product.

### Statistics

Data were analyzed with two-way analysis of variance (ANOVA) with genotype and sex as independent variables. Pre-pulse inhibition, learning tests, CLAMS, rota-rod and activity data were analyzed with two-way ANOVA for repeated measures with amplitude, trial number or time as the repeated measure. Multivariate ANOVA was applied when measuring different parameters derived from the same test. In simple tests with one variable only, the t-test was used. Barbering data and data from dominance test in IntelliCage were analyzed with a Chi-square test. Results are given as means ± SEM. Results were considered significant at *P* < 0.05.

## Results

### CD73 is expressed in subcortical structures of the forebrain and in meninges

**Figure 1:**
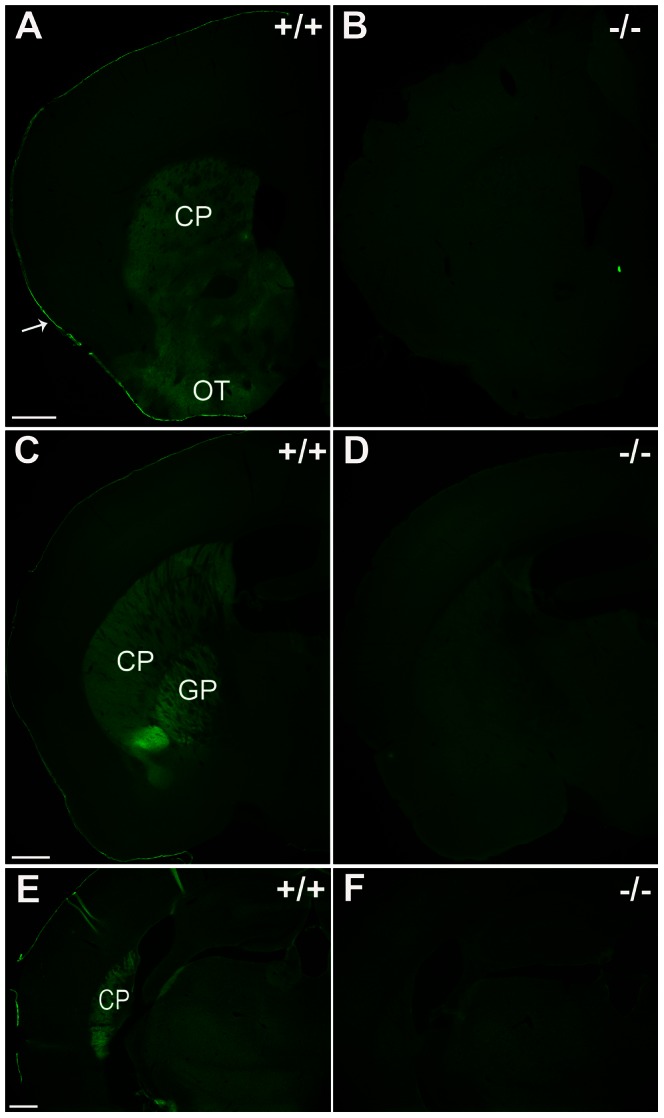
Immunohistochemical localization of CD73 in mouse brain. Coronal sections of WT (panels A, C and E) and CD73 deficient (panels B, D and F) mouse cerebrums stained with CD73 antibody. The specific CD73 staining is detected in caudoputamen (CP), olfactory tubercle (OT), globus pallidus (GP) and meninx (arrow). Scale bar 500 μm.

We studied the expression of CD73 using immunohistochemistry of wild-type and CD73 deficient mice. Specific and intense immunostaining was found in caudate and putamen that form the dorsal part of striatum (Fig. 1). The olfactory tubercle that belongs to ventral striatum was also found to clearly express CD73. Globus pallidus, a component of the basal ganglia core together with striatum, displayed intense expression. Furthermore, choroid plexus and meninges were found to be positive in CD73 immunohistochemistry.

### CD73 is the major 5’-nucleotidase hydrolyzing AMP in brain

**Figure 2:**
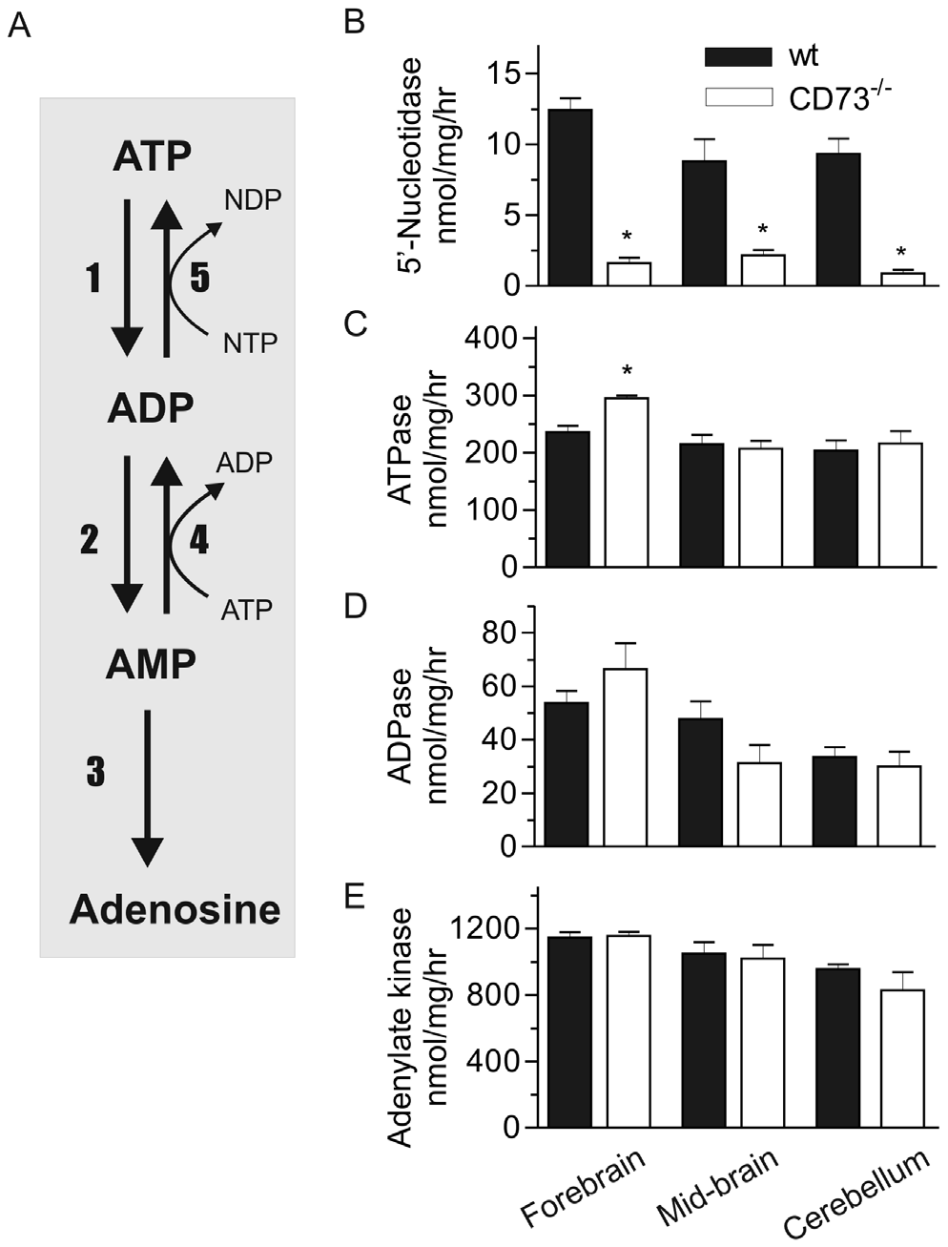
CD73 is the dominant 5’nucleotidase in the brain. (A) A scheme of the major extracellular nucleotide-converting pathways. The inactivating cascade is composed of NTPDase (1,2), and CD73/ecto-5’-nucleotidase (3), whereas the backward ATP-generating pathway is regulated by adenylate kinase (4) and NDP kinase (5). Brain lysates were isolated from forebrain, middle brain and cerebellum of wt and CD73 deficient mice and assayed for 5’-nucleotidase (B) ATPase (C), ADPase (D) and adenylate kinase (E) activities, as specified in Materials and Methods (mean±SEM; n = 4–5). *P<0.05 as compared with corresponding wt controls.

The duration and magnitude of purinergic signalling is known to be governed by a network of purine-converting ectoenzymes (Fig. 2A) abundantly expressed in various cells and tissues, including the brain [8], [20]. Since six other 5’nucleotidases in addition to CD73 have been described, we first analyzed the contribution of CD73 to the total AMP hydrolysis in the brain. Measurement of the rate of [^3^H]AMP breakdown revealed markedly decreased activity in all brain regions (forebrain, midbrain and cerebellum) in the absence of CD73 (Fig. 2B). Our data revealed that ecto-5’-nucleotidase/CD73 accounts for ∼85–95% of all AMP-hydrolyzing capability in the murine brain.

We then determined the activities of other key nucleotide converting enzymes in the different parts of brain in the wt and CD73 deficient mice (Figure 2C–E). To that end, the lysates from the forebrain, midbrain and cerebellum were incubated with saturating concentrations of [^3^H]ATP (Figure 2C) or [^3^H]ADP (Figure 2D). We found that all samples from both genotypes displayed significant nucleoside triphosphate diphosphohydrolase (NTPDase) activity with ATPase/ADPase ratio of ∼5. Interestingly, ATPase activity was slightly but significantly upregulated in the forebrain of the CD73 deficient mice. No other genotype-specific differences in the ATPase or ADPase activity were found in any part of the brain. The counteracting adenylate kinase activity was also studied by determining the phosphoryl transfer from ATP into [^3^H]AMP. We found that the adenylate kinase activities were similar in wt and CD73 deficient mice in all regions of the brain (Figure 2E). Together, these data suggest that a minor increase in the ATPase activity, probably as a compensatory mechanism, takes place in the forebrain of the CD73 deficient mice. However, despite of the dramatic decrease in the AMP hydrolysis, the activities of several other nucleotide-converting ecto-enzymes remain almost intact in the brain in the absence of CD73.

### CD73 deficient mice do not differ from wt mice in most behavioural tests

**Figure 3:**
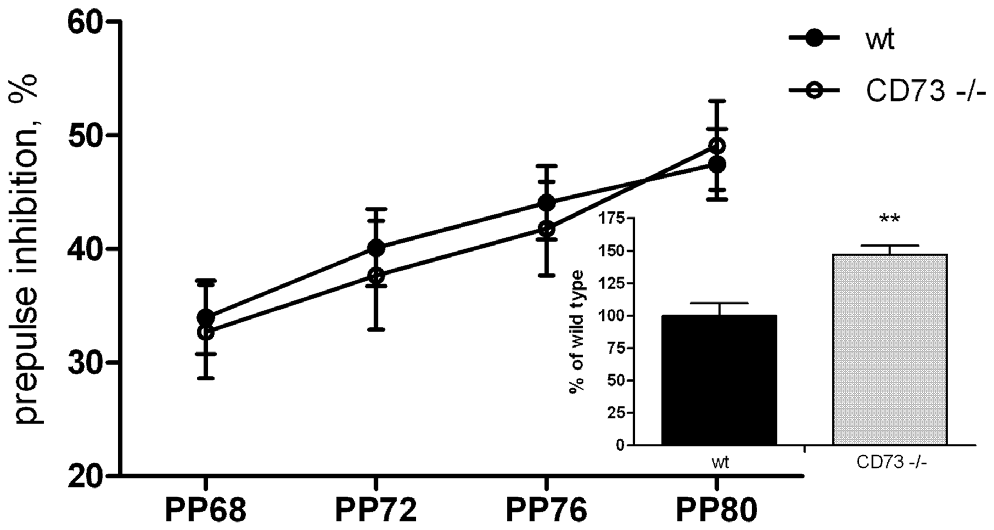
CD73 deficient and wt mice display similar pre-pulse inhibition but startle response is enhanced in CD73 deficient mice compared to wt mice. Percentage of pre-pulse inhibition averaged across all pre-pulse intensities. Insert bar graphs represent percentage of alteration of startle response without pre-pulse stimulus from the wild type animals’ startle response level (wt mice, n = 38; CD73 deficient mice, n = 29). The results from both sexes are combined, because there was no *sex* effect or *sex* X *genotype* interaction. Mean values are plotted with SEM, **p<0.01 t-test.

**Figure 4:**
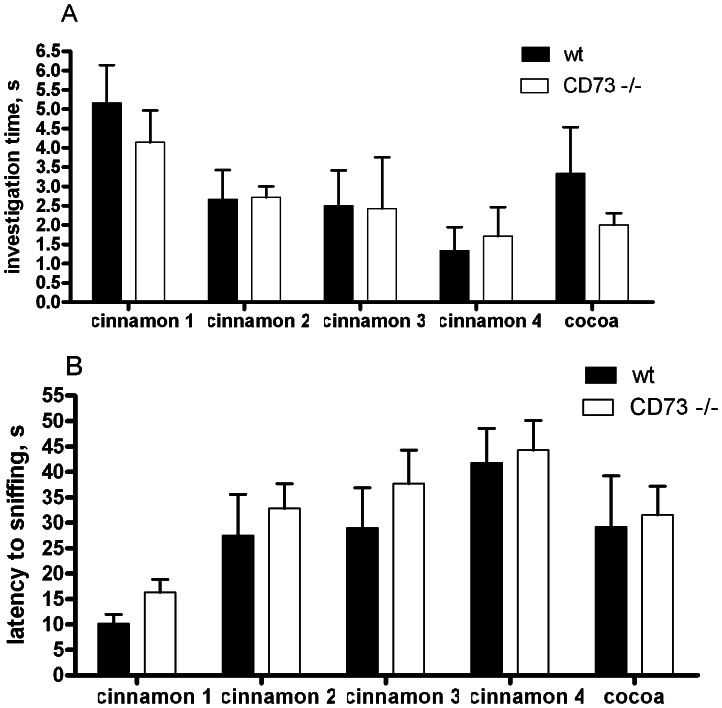
CD73 deficient mice do not differ from wt mice in olfactory discrimination test. (A) Latency to sniffing and (B) time spent in olfactory investigation of capsules with odors during four repeated trials (with cinnamon) and one trial of dishabituation (cocoa) (wt female mice, n = 6; CD73 deficient female mice, n = 7).

**Figure 5:**
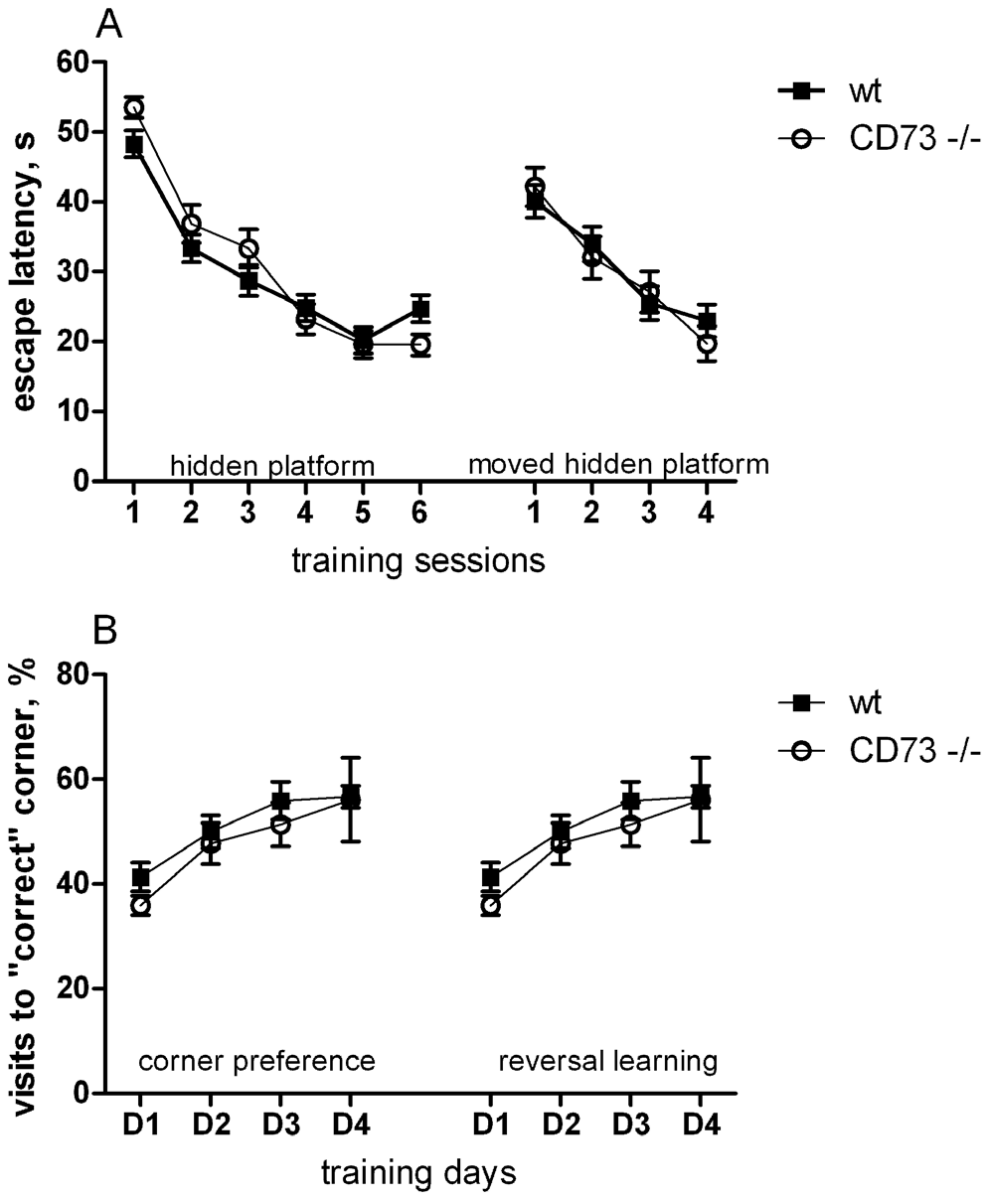
Learning in water maze and in IntelliCage is comparable in wt and CD73 deficient mice. (A) Escape latency to hidden platform during training and reversed training with moved platform in Morris water maze (wt mice, n = 38; CD73 deficient mice, n = 29). The results from both sexes are combined, because there was no *sex* effect or *sex* X *genotype* interaction. (B) Percentage of visits to the “correct” corner during corner preference learning and reversal learning in IntelliCage (CD73 deficient female mice, n = 9; wt female mice, n = 8).

Since enzymatically active CD73 was present in several brain regions, we speculated that the induction of the purinergic signalling through CD73-derived adenosine might be involved in controlling behaviour. To address this, we compared the wt and CD73 deficient mice in multiple behavioral tests performed both individually (summarized in Table 1) and also in the IntelliCage environment. For the assessment of anxiety level, three tests were performed: elevated plus-maze, open field and light-dark exploration. In these tests, the parameters reflecting anxiety-like behaviour were comparable in both genotypes (Table 1). Furthermore, in the forced swim test for the assessment of depression-like behaviour the wt and CD73 deficient mice demonstrated the same level of immobility in an inescapable situation (Table I). We found no significant genotype-specific differences in spontaneous alternation (Y maze), nociception (hot plate), motor coordination and motor learning, or in fear-conditioning. Pre-pulse inhibition did not differ either from that observed in the wt mice although the startle response to acoustic stimulus without pre-stimulus was increased in the CD73 deficient mice (Fig. 3), agreeing with novelty-induced hyperactivity in these mice (see below). Olfactory discrimination also appeared intact in the CD73 knockout mice (Fig. 4). The results from the corner preference learning tasks in the IntelliCage supported the lack of differences in spontaneous alternation and spatial learning as measured by Y-maze and water maze (Fig. 5). Thus, several behavioural parameters seem to be completely independent of CD73 activity.

**Table 1:** Summary of individual behavioural tests carried out in wt and CD73.

Test and parameters measured	wt	CD73 -/-	p-value
*Elevated plus maze*			
distance, cm	768.7±50.4	1009.2±70	p<0.01
open arm latency, s	104.2±19	117.5±22.9	ns
open entries, %	21.3±3.1	17.7±3.1	ns
open arm time, s	40.7±8	34.4±7.3	ns
rearings, n	16.5±1.3	15.8±1.5	ns
*Open field*			
distance, cm	4580.6±160.5	5568±213.6	p<0.01
distance in center, %	24.1±0.7	25.6±0.9	ns
latency to center, s	114.3±20.6	65.6±16.5	ns
time in center,s	320.4±18.9	323.7±24.9	ns
center entries, n	470.2±12.6	550.6±19.7	ns
rearing, s	380.4±24	429.9±33.1	ns
*Light-dark*			
distance, cm	1243.2±46.9	1258.3±55.4	ns
distance in light, %	38.6±1.4	39.2±1.4	ns
time in light, s	114.2±7.2	121.3±6	ns
rearing, s	40±2.2	34.3±2.9	ns
*Y-maze*			
spontaneous alternation, %	53.5±2.1	57.6±2.1	ns
*Hot plate*			
latency to reaction, s	13.7±0.8	12.8±0.6	ns
*Rota-Rod*			
latency to fall, first trial, s	216.6±17.4	193.6±20.6	ns
latency to fall, last trial, s	283±12.9	250.9±18.8	ns
*Fear-conditioning*			
contextual freezing, %	31±4.1	28.5±3.8	ns
cued freezing, %	38.2±3.4	38.4±4.3	ns
*Forced swim test*			
immobility time, s	161.2±11.3	134.9±16.2	ns

deficient mice.

### Locomotor activity is increased in CD73 deficient mice

**Figure 6:**
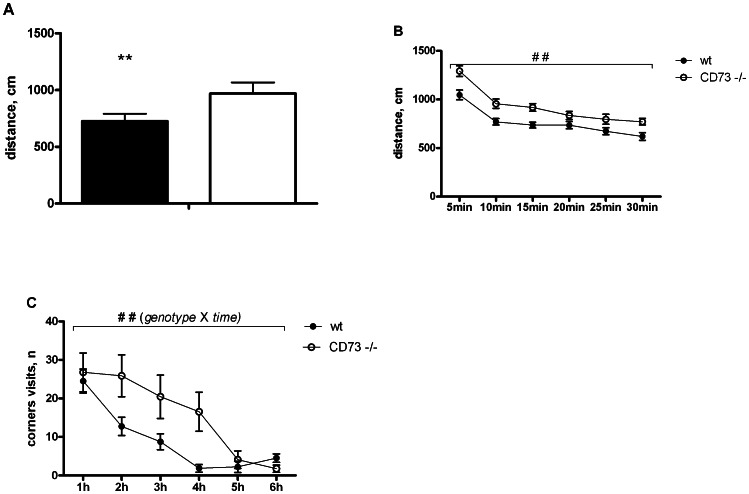
CD73 deficient mice demonstrate increased exploratory activity. The distances travelled by wt (n = 38 mice) and CD73 deficient (n = 29) mice in (A) the elevated plus maze and (B) open field tests. The results from both sexes are combined, because there was no *sex* effect or *sex* X *genotype* interaction. In Intellicage analyses the numbers of corner visits (mean ± SEM) are plotted between 1 and 6 hr (C). The IntelliCage data are from females only (CD73 deficient mice, n = 9; wt mice, n = 8). *p<0.05 t-test, **p<0.01 t-test, # p<0.05 ANOVA, ## p<0.01 ANOVA.

We found increased locomotor activity of the CD73 deficient mice in several assays. In the elevated plus maze, the CD73 deficient mice travelled longer distances than the wt mice (p = 0.0057; Fig. 6A).The mice lacking CD73 demonstrated increased locomotor activity in the open field arena when compared to the wt animals (p = 0.004; Fig. 6B).

Differences in the locomotor activity were also seen under undisturbed conditions in the home cages as revealed by IntelliCage studies. During the first phase of the study, when all mice had free access to all operant corners and water bottles, both groups performed about the same number of corner visits and visits with drinking behaviour. All mice had the same latency to the first corner visit and to visit of all four corners. The main difference in the activity started after 1 hour of novel environment exploration: the wt mice demonstrated progressive reduction in corner visits while the CD73 deficient mice remained active during the next 3 hours (F(5,75) = 3.87, p = 0.0036, two-way ANOVA, genotype and experimental time interaction) (Fig. 6C).

**Figure 7:**
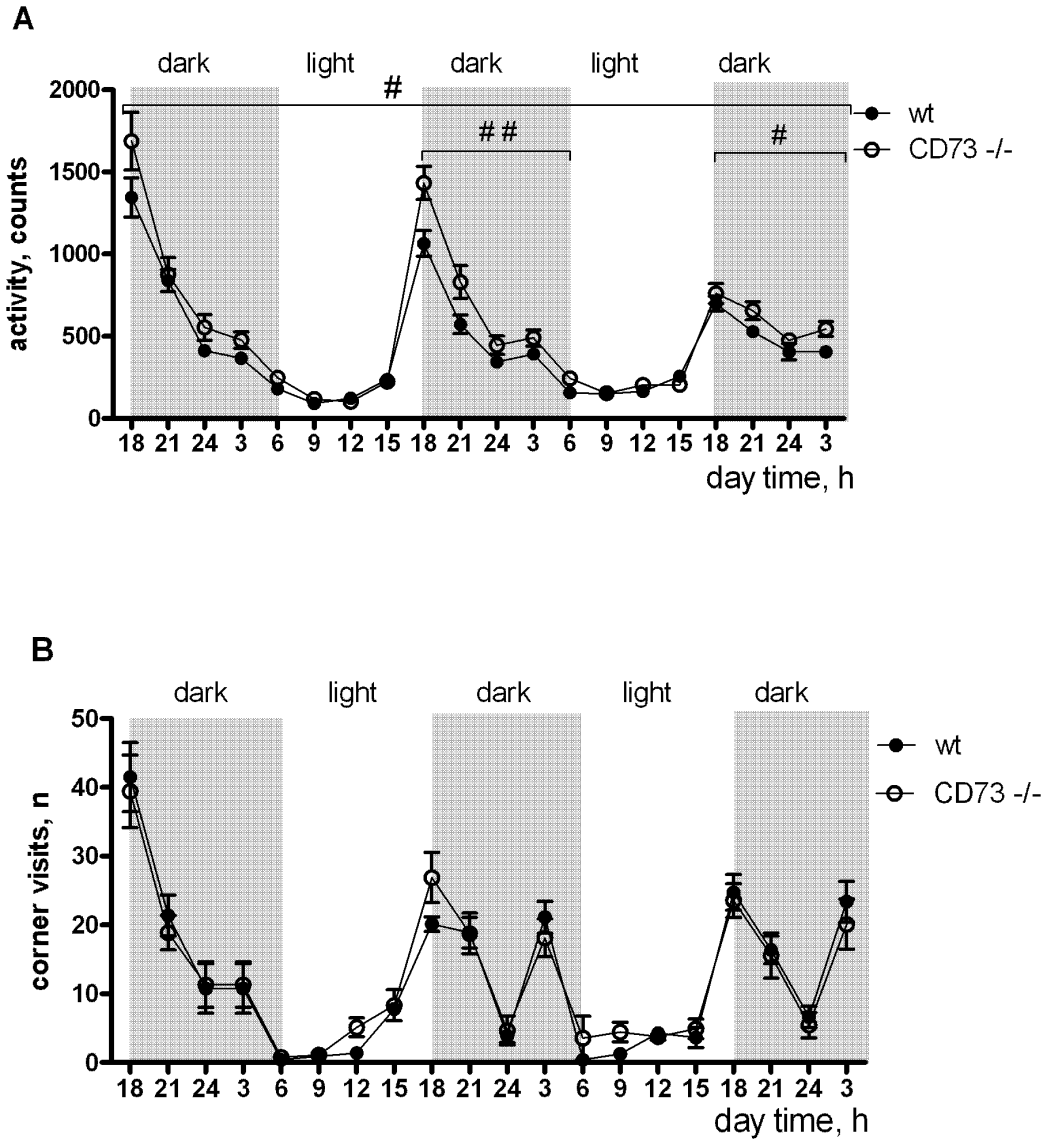
CD73 controls circadian changes in locomotor activity in isolated mice. (A) Locomotor activity presented as activity counts during 72 hr observation period, when the mice were separated in individual cages. These data are presented for both sexes together, because there was no *sex* effect or *sex* X *genotype* interaction (CD73 deficient mice, n = 25; wt mice, n = 30). (B) Locomotor activity measured as corner visits in IntelliCage. In IntelliCage analyses only females were used (CD73 deficient, n = 9; wt, n = 8). Mean values are plotted with SEM, # p<0.05 and ## p<0.01, repeated ANOVA.

Increased locomotor activity in the CD73 deficient mice was also observed in the circadian activity test when measured for 72 h using CLAMS (F(1,53) = 5.95, p = 0.02, ANOVA, main effect). The difference was observed during the dark period when the animals are naturally more active, whereas the light time activity was similar in both genotypes (Fig. 7A). However, the increased dark-period locomotor activity in the CD73 deficient mice was not observed in IntelliCage (Fig. 7B). One explanation for the different results could be a prolonged stress reaction during individual housing in small cages of CLAMS. We thus conclude that CD73 is normally needed to restrict exploratory activity.

### Social behaviour is altered in CD73 deficient mice

**Figure 8:**
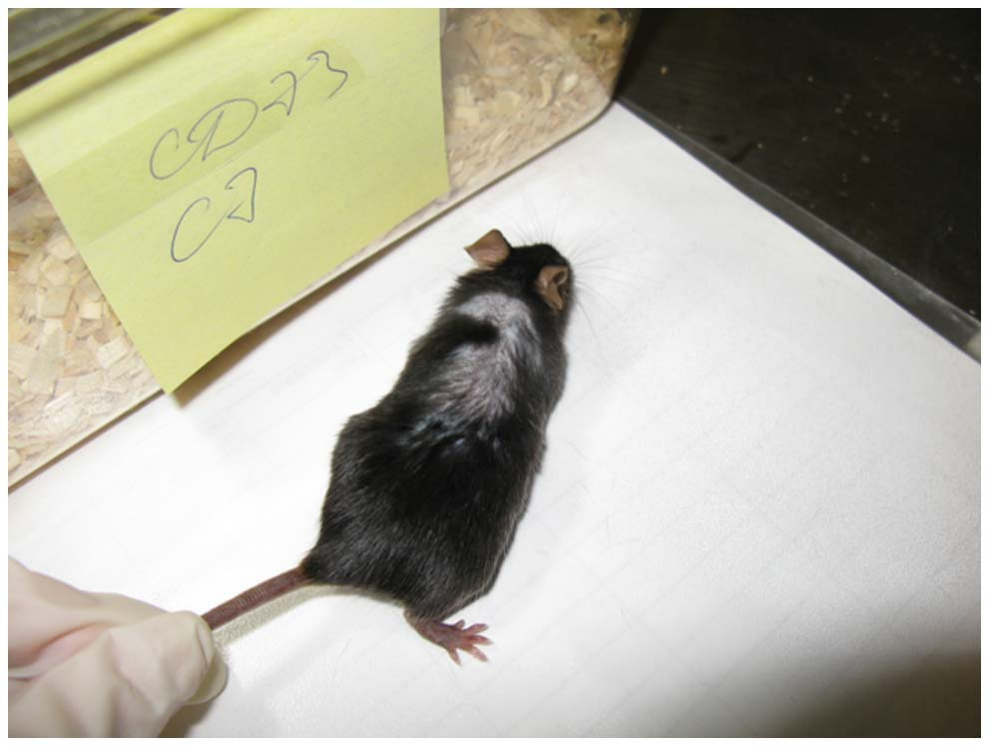
Mouse with hair loss (barbering). An example of a mouse with bald patches due to barbering.

Group-housed CD73 deficient mice showed a strong barbering habit, which manifested as big bald patches on the back (Fig. 8). Those patches were found in all mice except in one mouse (barber) per cage. In cages with the wt mice, barbering was not so evident (7 cages of knockout and 7 cages of wt mice; barbering observed in 7 cages for the knockout and in 2 cages for the wt mice; Chi-squared p = 0.0053). Regrowth of hair was observed within one week of individual housing for all animals.

**Figure 9:**
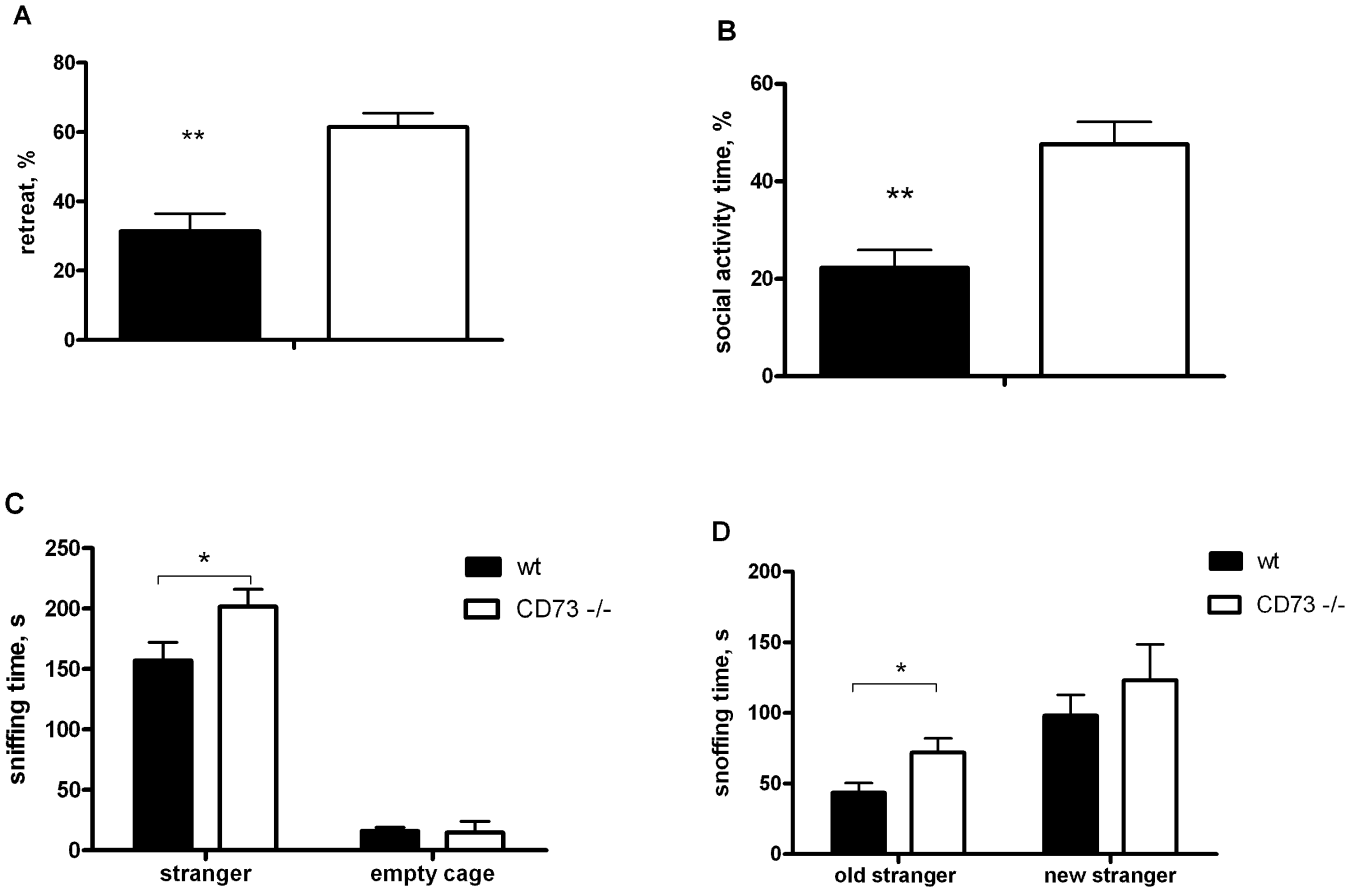
CD73 deficient mice display altered social behaviour. (A) Percentages of the times pushed out in the tube test (32 mice/group in both genotypes). (B) Percentage of the time spent in social activity towards the intruder in the resident-intruder test (wt mice, n = 15; CD73 deficient mice, n = 13). (C) Time spent in sniffing the stranger in the social novelty preference test, and (D) time spent in sniffing a new and an old stranger in the sociability test (wt, n = 16; CD73 deficient, n = 16). Mean values are plotted with SEM, *p<0.05 t-test, **p<0.01 t-test.

In the tube test, the CD73 deficient mice retreated from the tube in about 60% of the trials (p<0.0001) without significant sex effect (Fig. 9A). Nevertheless, the wt mice did not demonstrate any aggressive posture or fighting to push the CD73 deficient mice out from the tube. There was no difference between the genotypes in aggressiveness in the resident-intruder test either. At the same time, the CD73 deficient mice spent almost twice as much time as the wt mice (p<0.001) in social activity towards the intruder mice (Fig. 9B).

In the social novelty test, there was no difference in time and frequency of entries to the chambers with stranger mice during sociability or novelty preference sessions. All mice demonstrated preference to the chamber with a stranger mouse in comparison to an empty chamber, and subsequently to a chamber with a new stranger mouse in comparison to a familiar mouse (an old stranger). However, the CD73 deficient mice spent more time in sniffing new strangers (p = 0.0426) or old strangers (p = 0.0262) than the wt mice (Fig. 9C,D).

Behavioural assessment in IntelliCage allowed us to compare some behavioural characteristics obtained in individual standard tests with data obtained in social context with minimal human contact. In IntelliCage, the CD73 deficient mice showed more suppressive behaviour in drinking access competition allowing the wt mice to spend more time in the correct corner during drinking session. In group of wt animals 7 animals out of 8 demonstrated dominant behaviour while in CD73 deficient mice group only 1 out of 8 (Chi-squared p = 0.0016).

Altogether, these tests show that in the absence of CD73 the mice display increased social activities but decreased social dominance.

## Discussion

### CD73 is the major 5′-nucleotidase generating adenosine in brain

CD73 (ecto-5’-nucelotidase) is one of the several enzymes possessing 5’-nucleotidase activity. Other 5’-nucleotidases are structurally different from CD73 and expressed either in the cytoplasm or in mitochondria. 5’-nucleotidases are known to be widely expressed in the nervous system [20], [21]. Based on enzyme cytochemistry, 5′-nucleotidases are predominantly associated with glial cells in the mature nervous system, while during development and regeneration they are active in the synaptic cleft. However, the expression analyses have been mainly based on enzyme cytochemistry that does not allow discrimination between different 5’-nucleotidases [22]. Thus, our immunohistochemical analysis using wild-type and CD73 deficient mice clearly discriminates the expression of CD73 from other 5’-nucleotidases in the brain. The prominent expression found in the basal ganglia core is in agreement with previous work based on detection of ectonucleotidase activities [21], although assays based on the enzyme activity suggest a wider distribution compared to our results. With the exception of vascular expression of CD73 in the nervous system [9], [23], immunohistochemical localization in brain based on specific anti-CD73 antibodies has remained unclear. Our current immunohistochemical findings indicate that within the brain parenchyma, the CD73 protein is present in the subcortical regions, especially in striatum and globus pallidus. Moreover, our functional analyses using CD73 deficient mice reveal that CD73 confers the majority of the 5′-nucleotidase activity in the brain.

### CD73 regulates exploratory locomotor activity

Adenosine has been shown to be involved in the regulation of wakefulness, sleep, learning and memory, fear, anxiety, motor functions as revealed by pharmacological or genetic tools [6], [24]-[26]. Therefore, we subjected the CD73 deficient mice to a broad battery of behavioural tests.

Hyperactivity was revealed in the CD73 deficient mice using several standard individual tests, such as open field, elevated plus maze and long-term monitoring using the CLAMS system. Increased locomotion in the absence of CD73 was also observed in IntelliCage, which allows long-term monitoring in normal social environment without stress of social isolation or human interference. Moreover, the results from IntelliCage suggest that the CD73 deficient mice display altered exploratory behaviour rather than simple hyperactivity. At the beginning of the experiment all animals explored a novel environment quite actively and then progressively decreased their activity with time. Four hours in a novel environment was enough for the wt mouse to become calm, while the CD73 deficient animals were still very active in their exploration.

Our finding of enhanced locomotor activity is in contrast with a recent study characterizing a different line of CD73 deficient mice [27], where no hyperactivity in novel environment was observed. Moreover, those mice were characterized as exhibiting superior working memory and habituation along with reduced performance in rota-rod task. Neither of these findings was confirmed by our experiments. Differences in generation of the mutant mice, small sample size and different environmental conditions (e.g. individual housing) could explain these differences and warrant further studies regarding the effect of various environmental challenges on the phenotype of CD73 mice.

The observation of hyperactivity in CD73 deficient mice agrees well with the finding that CD73 is a major 5′-nucleotidase generating adenosine in brain since adenosine is a global regulator of neuronal activity in brain, causing a basal inhibitory tone of behavioural activity [6]. Lack of CD73 thus causes a deficiency in adenosine-dependent signalling that cannot be compensated by other 5′-nucleotidases of brain. High expression of CD73 in the striatum suggests involvement of A2A receptors in mediating the observed locomotor phenotype. Indeed, adenosine A2A receptor antagonists produce motor effects in animal models [28], [29] and there is considerable interaction between adenosine and dopamine receptors in the striatum that subsequently influences spontaneous locomotor activity [28], [30]–[32].

### Modulation of social behaviour in CD73 deficient mice

We observed increased spontaneous and circadian activity in the CD73 deficient mice in individual testing, but not when the mice lived in groups. This suggests that behaviour of the CD73 deficient mice is strongly dependent on their social environment. Therefore, we analyzed their social interactions more carefully. The CD73 deficient mice showed intensive barbering behaviour. In group-housed mice barbering is an intensive form of hetero-grooming [33]. Barbering can represent social hierarchy in an animal group with dominant animals acting as barbers. Our findings thus suggest that the CD73 deficient animals may have a clearer social organisation.

The tube test measuring dominant behaviour demonstrated that the CD73 deficient mice were less persistent than wt mice in pushing their opponent of the opposite genotype out from the tube. The resident-intruder test supported the idea about more intensive social behaviour in the absence of CD73. The CD73 deficient mice spent visibly more time in different kinds of social interactions, such as hetero-grooming, sniffing and observing the intruder without fighting and clear aggressiveness. In the assessments of the sociability level and preference to social novelty [18], [34], the CD73 deficient mice spent more time in direct sniffing of the first stranger. This suggests that social contacts and interactions initiated in the absence of CD73 are more prolonged and persistent than those of the wt mice. The increased social activity found in the CD73 deficient mice is probably related to and well in line with the enhanced exploratory locomotor activity found in these mice in open field and IntelliCage tests.

It is noteworthy that the mice lacking A2A or A1 receptors display enhanced anxiety and aggression [35], [36]. Moreover, the mice selectively bred for maternal aggression exhibit significantly enhanced levels of adenosine A1 receptors [37]. These findings together with our observations strongly suggest involvement of adenosine in modulation of social behaviour.

### Preserved sensory and memory functions in CD73 deficient mice

Mature retina and olfactory bulbs are rare examples of tissues in which spontaneous synaptic turnover occurs in adulthood. Schoen and Kreutzberg have shown that this persistent synaptic change in olfactory bulbs involves expression of 5’nucleotidases at synaptic contacts in adult rats [22], [38]. Olfaction is a critical function for normal adaptation to environment, social behaviour and social self-determination of mammals. Rodents use it to explore novel environment, and unfamiliar conspecific pups learn to find their mothers and later in adulthood partners for mating. Abnormalities of the olfactory system can thus affect social learning [39], social behaviour by itself and exploratory strategy [40], [41]. In addition, neurological/neuropsychiatric disorders such as Alzheimer’s [42], [43], Parkinson’s [44], [45] and Huntington’s [46] diseases are associated with olfactory deficit. Importantly, we did not see any evidence about abnormalities in the olfactory system in the CD73 deficient animals. This suggests that their abnormalities in locomotor activity and social behaviour are not connected to the olfactory system and that other, non-CD73 5’-nucleotidase species are operational in olfactory bulbs.

CD73 was very recently reported to inhibit nociception due to adenosine production in nociceptive circuits. The function in nociception was revealed under sensitized conditions but not under basal conditions [47]. These findings agree with our analysis that did not reveal a genotype difference in nociception under basal conditions.

We were not able to detect any difference in learning and memory (assessed by spatial water maze, fear conditioning and IntelliCage) in our CD73 deficient mice, although evidence exists for purinergic signalling to be implicated in learning and memory [24], [27], [48], [49].

### Concluding remarks

Our results open up new avenues for understanding the specific role of CD73/ecto-5’-nucelotidase, over the other 5’-nucleotidases, in the brain. They reveal that locomotor activity and social contacts are behavioural patterns which are normally controlled by CD73 activity. It is worth noting that changes in these behavioural patterns belong to endophenotypes (for the endophenotype concept, see [50]) of common behavioural disorders, such as schizophrenia [51] and autism [52]. Furthermore, changes in adenosinergic signalling have been previously linked to behavioural disorders, including schizophrenia [53]. The finding that CD73 is a major regulator of adenosinergic signalling in brain may provide a tool for pharmaceutical intervention in behavioural disorders.

Our finding that CD73 is prominently expressed in striatum agrees very well with the behavioural analysis since striatum is best known for its role in planning and regulation of movement pathways. It is also worth noting that striatal dysfunction is associated to Parkinsońs and Huntingtońs diseases characterized by movement disorders. Furthermore, we have found CD73 expression in the olfactory tubercle within the brain, an area regulating social behaviour and locomotion [54]. However, further work is warranted to reveal how the CD73/adenosine-dependent signalling in these locations is transmitted to the complex receptor system of purinergic signalling including different adenosine receptors and their interactions with dopamine and glutamate receptors.

## References

[1] Hasko G, Linden J, Cronstein B, Pacher P (2008) Adenosine receptor: therapeutic aspects for inflammatory and immune diseases. Nat Rev Drug Discov 7: 759–770. 18758473 10.1038/nrd2638 PMC2568887

[2] Boison D, Chen JF, Fredholm BB (2010) Adenosine signaling and function in glial cells. Cell Death Differ 17: 1071–1082. 19763139 10.1038/cdd.2009.131 PMC2885470

[3] Jones BE (2009) Glia, adenosine, and sleep. Neuron 61: 156–157. 19186158 10.1016/j.neuron.2009.01.005

[4] Sebastião AM, Ribeiro JA (2009) Tuning and fine-tuning of synapses with adenosine. Curr Neuropharmacol 7: 180–194. 20190960 10.2174/157015909789152128 PMC2769002

[5] Ribeiro JA, Sebastião AM, de Mendonça A (2003) Adenosine receptors in the nervous system: pathophysiological implications. Prog Neurobiol 68: 377–392. 10.1016/s0301-0082(02)00155-7 12576292

[6] Dunwiddie TV, Masino SA (2001) The role and regulation of adenosine in the central nervous system. Annu Rev Neurosci 24: 31–55. 11283304 10.1146/annurev.neuro.24.1.31

[7] Eltzschig HK, Sitkovsky MV, Robson SC (2012) Purinergic signaling during inflammation. N Engl J Med 367: 2322–2333. 23234515 10.1056/NEJMra1205750 PMC3675791

[8] Yegutkin GG (2008) Nucleotide- and nucleoside-converting ectoenzymes: Important modulators of purinergic signalling cascade. Biochim Biophys Acta 1783: 673–694. 18302942 10.1016/j.bbamcr.2008.01.024

[9] Niemelä J, Ifergan I, Yegutkin G, Jalkanen S, Prat A, et al (2008) Interferon-B regulates CD73 and adenosine expression at the blood-brain-barrier. Eur J Immunol 38: 2718–2726. 18825744 10.1002/eji.200838437

[10] Kiss J, Jalkanen S, Fulop F, Savunen T, Salmi M (2008) Ischemia-reperfusion injury is attenuated in VAP-1-deficient mice and by VAP-1 inhibitors. Eur J Immunol 38: 3041–3049. 18991279 10.1002/eji.200838651

[11] Koszalka P, Ozuyaman B, Huo YQ, Zernecke A, Flogel U, et al (2004) Targeted disruption of cd73/ecto-5 '-nucleotidase alters thromboregulation and augments vascular inflammatory response. Circ Res 95: 814–821. 15358667 10.1161/01.RES.0000144796.82787.6f

[12] Thompson LF, Eltzschig HK, Ibla JC, Van De Wiele CJ, Resta R, et al (2004) Crucial role for ecto-5'-nucleotidase (CD73) in vascular leakage during hypoxia. J Exp Med 200: 1395–1405. 15583013 10.1084/jem.20040915 PMC1237012

[13] Võikar V, Polus A, Vasar E, Rauvala H (2005) Long-term individual housing in C57BL/6J and DBA/2 mice: assessment of behavioral consequences. Genes Brain Behav 4: 240–252. 15924556 10.1111/j.1601-183X.2004.00106.x

[14] Võikar V, Vasar E, Rauvala H (2004) Behavioral alterations induced by repeated testing in C57BL/6J and 129S2/Sv mice: implications for phenotyping screens. Genes Brain Behav 3: 27–38. 14960013 10.1046/j.1601-183x.2003.0044.x

[15] Yegutkin GG, Henttinen T, Jalkanen S (2001) Extracellular ATP formation on vascular endothelial cells is mediated by ecto-nucleotide kinase activities via phosphotransfer reactions. FASEB J 15: 251–260. 11149913 10.1096/fj.00-0268com

[16] Wrenn C, Harris A, Saavedra M, Crawley J (2003) Social transmission of food preference in mice: methodology and application to galanin-overexpressing transgenic mice. Behav Neurosci 117: 21–31. 12619904

[17] Messeri P, Eleftheriou B, Oliverio A (1975) Dominance behavior: a phylogenetic analysis in the mouse. Physiol Behav 14: 53–58. 1171474 10.1016/0031-9384(75)90141-9

[18] Nadler J, Moy S, Dold G, Trang D, Simmons N, et al (2004) Automated apparatus for quantitation of social approach behaviors in mice. Genes Brain Behav 3: 303–314. 15344923 10.1111/j.1601-183X.2004.00071.x

[19] Krackow S, Vannoni E, Codita A, Mohammed AH, Cirulli F, et al (2010) Consistent behavioral phenotype differences between inbred mouse strains in the IntelliCage. Genes Brain Behav 9: 722–731. 20528956 10.1111/j.1601-183X.2010.00606.x

[20] Zimmermann H (1996) Biochemistry, localization and functional roles of ecto-nucleotidases in the nervous system. Prog Neurobiol 49: 589–618. 8912394 10.1016/0301-0082(96)00026-3

[21] Langer D, Hammer K, Koszalka P, Schrader J, Robson S, et al (2008) Distribution of ectonucleotidases in the rodent brain revisited. Cell Tissue Res 334: 199–217. 18843508 10.1007/s00441-008-0681-x

[22] Schoen S, Kreutzberg G (1995) Evidence that 5'-nucleotidase is associated with malleable synapses - an enzyme cytochemical investigation of the olfactory bulb of adult rats. Neuroscience 65: 37–50. 7753405 10.1016/0306-4522(94)00469-l

[23] Mills J, Thompson L, Mueller C, Waickman A, Jalkanen S, et al (2008) CD73 is required for efficient entry of lymphocytes into the central nervous system during experimental autoimmune encephalomyelitis. Proc Natl Acad Sci U S A 105: 9325–9330. 18591671 10.1073/pnas.0711175105 PMC2453691

[24] Burnstock G, Krugel U, Abbracchio MP, Illes P (2011) Purinergic signalling: from normal behaviour to pathological brain function. Prog Neurobiol 95: 229–274. 21907261 10.1016/j.pneurobio.2011.08.006

[25] Shen HY, Chen JF (2009) Adenosine A(2A) receptors in psychopharmacology: modulators of behavior, mood and cognition. Curr Neuropharmacol 7: 195–206. 20190961 10.2174/157015909789152191 PMC2769003

[26] Yaar R, Jones MR, Chen JF, Ravid K (2005) Animal models for the study of adenosine receptor function. J Cell Physiol 202: 9–20. 15389588 10.1002/jcp.20138

[27] Zlomuzica A, Burghoff S, Schrader J, Dere E (2012) Superior working memory and behavioural habituation but diminished psychomotor coordination in mice lacking the ecto-5'-nucleotidase (CD73) gene. Purinergic Signal in press. 10.1007/s11302-012-9344-1 PMC3646114 23274765

[28] Salamone JD, Ishiwari K, Betz AJ, Farrar AM, Mingote SM, et al (2008) Dopamine/adenosine interactions related to locomotion and tremor in animal models: possible relevance to parkinsonism. Parkinsonism Relat Disord 14 Suppl 2S130–134. 18585081 10.1016/j.parkreldis.2008.04.017 PMC2806674

[29] Florio C, Rosati AM, Traversa U, Vertua R (1997) Inhibitory and excitatory effects of adenosine antagonists on spontaneous locomotor activity in mice. Life Sci 60: 1477–1486. 9126868 10.1016/s0024-3205(97)00099-4

[30] Xie X, Ramkumar V, Toth LA (2007) Adenosine and dopamine receptor interactions in striatum and caffeine-induced behavioral activation. Comp Med 57: 538–545. 18246865

[31] Ferre S, Fredholm BB, Morelli M, Popoli P, Fuxe K (1997) Adenosine-dopamine receptor-receptor interactions as an integrative mechanism in the basal ganglia. Trends Neurosci 20: 482–487. 9347617 10.1016/s0166-2236(97)01096-5

[32] Salmi P, Chergui K, Fredholm BB (2005) Adenosine-dopamine interactions revealed in knockout mice. J Mol Neurosci 26: 239–244. 16012197 10.1385/JMN:26:2-3:239

[33] Kalueff A, Minasyan A, Keisala T, Shah Z, P T (2006) Hair barbering in mice: implications for neurobehavioural research. Behav Processes 71: 8–15. 16236465 10.1016/j.beproc.2005.09.004

[34] Moy S, Nadle rJ, Young N, Nonneman R, Grossman A, et al (2009) Social approach in genetically engineered mouse lines relevant to autism. Genes Brain Behav 8: 129–142. 19016890 10.1111/j.1601-183X.2008.00452.x PMC2659808

[35] Ledent C, Vaugeois JM, Schiffmann SN, Pedrazzini T, El Yacoubi M, et al (1997) Aggressiveness, hypoalgesia and high blood pressure in mice lacking the adenosine A2a receptor. Nature 388: 674–678. 9262401 10.1038/41771

[36] Gimenez-Llort L, Fernandez-Teruel A, Escorihuela RM, Fredholm BB, Tobena A, et al (2002) Mice lacking the adenosine A1 receptor are anxious and aggressive, but are normal learners with reduced muscle strength and survival rate. Eur J Neurosci 16: 547–550. 12193199 10.1046/j.1460-9568.2002.02122.x

[37] Gammie SC, Auger AP, Jessen HM, Vanzo RJ, Awad TA, et al (2007) Altered gene expression in mice selected for high maternal aggression. Genes Brain Behav 6: 432–443. 16939635 10.1111/j.1601-183X.2006.00271.x PMC1994650

[38] Schoen S, Kreutzberg G (1997) 5'-nucleotidase enzyme cytochemistry as a tool for revealing activated glial cells and malleable synapses in CNS development and regeneration. Brain Res Brain Res Protoc 1: 33–43. 9385045 10.1016/s1385-299x(96)00006-2

[39] Sanchez-Andrade G, Kendrick K (2009) The main olfactory system and social learning in mammals. Behav Brain Res 200: 323–335. 19150375 10.1016/j.bbr.2008.12.021

[40] Stork O, Welzl H, Cremer H, Schachner M (1997) Increased intermale aggression and neuroendocrine response in mice deficient for the neural cell adhesion molecule (NCAM). Eur J Neurosci 9: 1117–1125. 9215693 10.1111/j.1460-9568.1997.tb01464.x

[41] Vinkers CH, Breuer ME, Westphal KG, Korte SM, Oosting RS, et al (2009) Olfactory bulbectomy induces rapid and stable changes in basal and stress-induced locomotor activity, heart rate and body temperature responses in the home cage. Neuroscience 159: 39–46. 19136045 10.1016/j.neuroscience.2008.12.009

[42] Murphy C, Solomon E, Haase L, Wang M, Morgan C (2009) Olfaction in aging and Alzheimer's disease: event-related potentials to a cross-modal odor-recognition memory task discriminate ApoE epsilon4+ and ApoE epsilon 4- individuals. Ann N Y Acad Sci 1170: 647–657. 19686207 10.1111/j.1749-6632.2009.04486.x PMC4575288

[43] Wesson D, Levy E, Nixon R, Wilson D (2010) Olfactory dysfunction correlates with amyloid-beta burden in an Alzheimer's disease mouse model. J Neurosci 30: 505–514. 20071513 10.1523/JNEUROSCI.4622-09.2010 PMC2826174

[44] Karpa M, Gopinath B, Rochtchina E, Wang JJ, Cumming R, et al (2010) Prevalence and neurodegenerative or other associations with olfactory impairment in an older community. J Aging Health 22: 154–168. 20133956 10.1177/0898264309353066

[45] Oka H, Toyoda C, Yogo M, Mochio S (2010) Olfactory dysfunction and cardiovascular dysautonomia in Parkinson's disease. J Neurol 257: 969–976. 20119648 10.1007/s00415-009-5447-1

[46] Lazic S, Goodman A, Grote H, Blakemore C, Morton A, et al (2007) Olfactory abnormalities in Huntington's disease: decreased plasticity in the primary olfactory cortex of R6/1 transgenic mice and reduced olfactory discrimination in patients. Brain Res 1151: 219–226. 17400200 10.1016/j.brainres.2007.03.018

[47] Sowa N, Taylor-Blake B, Zylka M (2010) Ecto-5′-Nucleotidase (CD73) inhibits nociception by hydrolyzing AMP to adenosine in nociceptive circuits. J Neurosci 30: 2235–2244. 20147550 10.1523/JNEUROSCI.5324-09.2010 PMC2826808

[48] Bonan CD, Roesler R, Pereira GS, Battastini AM, Izquierdo I, et al (2000) Learning-specific decrease in synaptosomal ATP diphosphohydrolase activity from hippocampus and entorhinal cortex of adult rats. Brain Res 854: 253–256. 10784132 10.1016/s0006-8993(99)02300-8

[49] Bonan CD, Dias MM, Battastini AM, Dias RD, Sarkis JJ (1998) Inhibitory avoidance learning inhibits ectonucleotidases activities in hippocampal synaptosomes of adult rats. Neurochem Res 23: 977–982. 9690740 10.1023/a:1021084422228

[50] Gould TD, Gottesman II (2006) Psychiatric endophenotypes and the development of valid animal models. Genes Brain Behav 5: 113–119. 16507002 10.1111/j.1601-183X.2005.00186.x

[51] Powell CM, Miyakawa T (2006) Schizophrenia-relevant behavioral testing in rodent models: a uniquely human disorder? Biol Psychiatry 59: 1198–1207. 16797265 10.1016/j.biopsych.2006.05.008 PMC3928106

[52] Crawley JN (2007) Mouse behavioral assays relevant to the symptoms of autism. Brain Pathol 17: 448–459. 17919130 10.1111/j.1750-3639.2007.00096.x PMC8095652

[53] Gotoh L, Mitsuyasu H, Kobayashi Y, Oribe N, Takata A, et al (2009) Association analysis of adenosine A1 receptor gene (ADORA1) polymorphisms with schizophrenia in a Japanese population. Psychiatr Genet 19: 328–335. 19820430 10.1097/YPG.0b013e3283328e26

[54] Ikemoto S (2007) Dopamine reward circuitry: two projection systems from the ventral midbrain to the nucleus accumbens-olfactory tubercle complex. Brain Res Rev 56: 27–78. 17574681 10.1016/j.brainresrev.2007.05.004 PMC2134972

